# Severe and isolated headache associated with hypertension as unique clinical presentation of posterior reversible encephalopathy syndrome

**DOI:** 10.1186/1471-2431-14-190

**Published:** 2014-07-25

**Authors:** Gregorio Paolo Milani, Alberto Edefonti, Giacomo Tardini, Elisa Arturi, Claudia Maria Cinnante, Emanuela Anna Laicini, Ernesto Leva, Alberto Maria Cappellari, Carlo Agostoni, Emilio Filippo Fossali

**Affiliations:** 1 Foundation IRCCS Ca’ Granda, Ospedale Maggiore Policlinico, Pediatric Emergency Department, Milan, Italy; 2 Foundation IRCCS Ca’ Granda, Ospedale Maggiore Policlinico, Pediatric Nephrology and Dialysis Department, Milan, Italy; 3 Foundation IRCCS Ca’ Granda, Ospedale Maggiore Policlinico, Neuroradiology Department, Milan, Italy; 4 Foundation IRCCS Ca’ Granda, Ospedale Maggiore Policlinico, Pediatric Surgery Department, Milan, Italy; 5 Foundation IRCCS Ca’ Granda, Ospedale Maggiore Policlinico, Department of Neuroscience and Mental Health, Milan, Italy; 6 Department of Clinical Sciences and Community Health, University of Milan, IRCCS Ospedale Maggiore Policlinico, Pediatric Clinic 2, Milan, Italy

**Keywords:** Posterior reversible encephalopathy syndrome, Arterial hypertension, Headache

## Abstract

**Background:**

Posterior reversible encephalopathy syndrome is a potentially reversible clinicoradiologic syndrome characterized by headache, mental confusion, visual disturbances and seizures associated with posterior cerebral lesions on radiological imaging. Prompt treatment of this condition is mandatory to avoid severe irreversible complications.

**Case presentation:**

We report a 9-year-old boy with arterial hypertension and headache as unique clinical presentation of posterior reversible encephalopathy syndrome.

**Conclusions:**

Severe and isolated headache associated with arterial hypertension can be the unique clinical presentation of posterior reversible encephalopathy syndrome. This syndrome must be considered even in absence of all typical symptoms to prevent the progression of a potentially life threatening condition.

## Background

Posterior reversible encephalopathy syndrome (PRES), first described by Hinchey *et al*. in 1996 [1], is a clinicoradiological condition presenting with headache, seizures, alterations of conscious level and loss of vision, accompanied by characteristic Magnetic Resonance Imaging (MRI) findings. Both the neurological and radiological features are potentially reversible [2]. Nevertheless several complications as status epilepticus, intracranial hemorrhage, and massive ischemic infarction have been reported in association with this syndrome [3,4]. The removal of the underlying causes is essential to prevent long term sequelae [3,4].

We report a child with PRES presenting as hypertension and severe headache, with rapid clinical and neuroimaging normalization after a prompt treatment.

## Case presentation

A previously normotensive 9-year-old boy was admitted to the Pediatric Emergency Department with bilateral periorbital edema and gross hematuria for 24 hours.

20 days before, he had experienced an episode of mild sore throat spontaneously recovered.

On admission general conditions, neurological and visual examinations were normal except for periorbital edemas. Axillary temperature was 36.9°C, oxygen saturation 98%, heart rate 88 beats per minute, body weight 35 kg (two more than the previous week), and body height 1.350 m. Blood pressure was increased with systolic values ranging between 125 and 130 mmHg and diastolic values ranging between 80 and 85 mmHg (stage 1 hypertension: 95^th^ percentile to the 99^th^ percentile for gender, age, and height plus 5 mmHg [5]). Blood and urine investigations were consistent for acute renal insufficiency due to a nephritic-nephrotic syndrome (Table 1).

**Table 1:** Blood and urine exams on admission

	**Value**	**Reference range**
*Blood exams*		
Red blood cells	4.2 x 10^6^/mmc	3.9 – 5.2 x 10^6^/mmc
Hematocrit	39%	34.5% - 42.5%
Hemoglobin	12.2 g/dL	10.5 - 14.5 g/dL
C-reactive protein	0.2 mg/dL	< 0.5 mg/dL
pH	7.39	7.38 -7.42
Sodium	138 mEq/L	135-145 mEq/L
Potassium	4.0 mEq/L	3.5 – 5.0 mE/L
Ionized calcium	1.20 mmol/L	1.12 – 1.32 mmol/L
Creatinine	1.1 mg/dL	< 0.7 mg/dL
Nitrogen urea	100 mg/dL	15 – 40 mg/dL
Albumin	3.0 g/dL	3.5 – 5.0 g/dL
Complement C3	17 mg/dL	86 – 184 mg/dL
Antistreptolysin titer	315 U/ml	0 – 200 U/ml
*Urine exams*		
Protein/creatinin ratio	3.5 mg/mg	< 0.4 mg/mg
Red cells number	55 per high-power field	< 5 per high-power field
Hyaline casts on sediment	Present	Absent

Since the 2^nd^ day of hospitalization, the patient was treated with one bolus of methylprednisolone (15 mg/Kg) per day associated with strict blood pressure monitoring.

**Figure 1:**
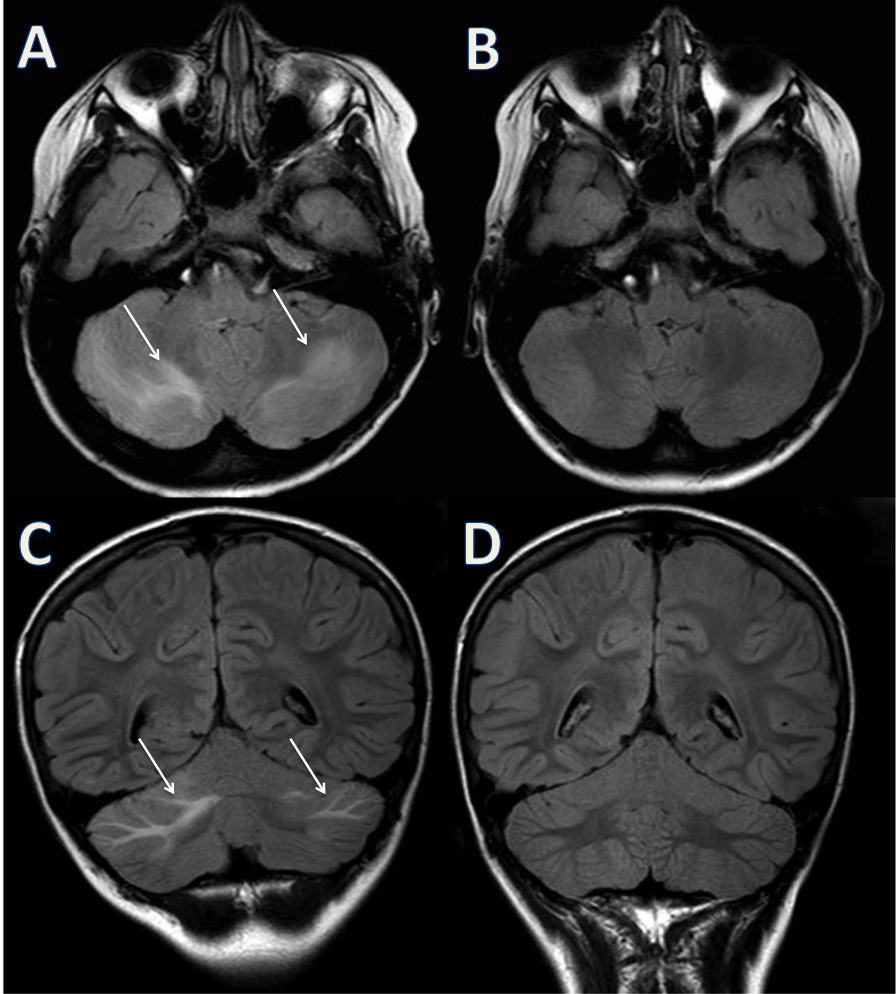
**Brain MRI cerebellar features.** MRI fluid attenuated inversion recovery (FLAIR) T2 images on axial **(A)** and coronal planes **(C)** showing high signal intensities in cerebellar white matter (indicated by the arrows). Follow up after 6 days showing a complete resolution of the lesions on axial **(B)** and coronal planes **(D)**.

**Figure 2:**
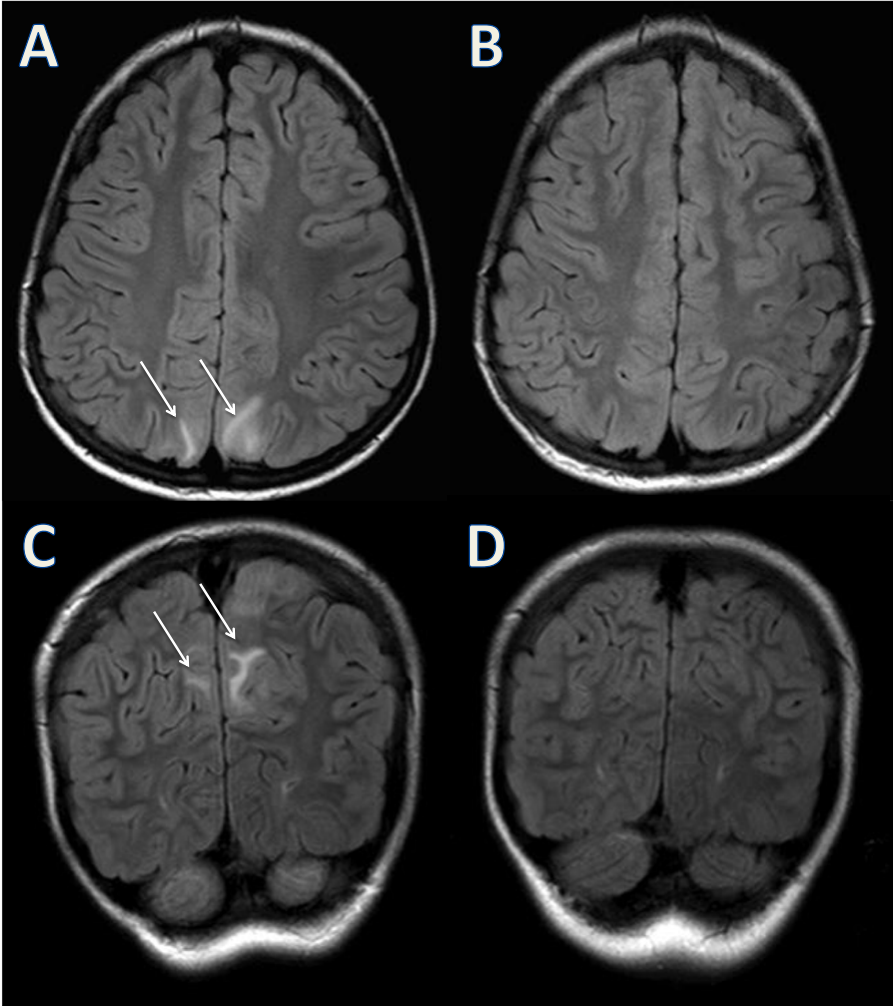
**Brain MRI parietal features.** MRI fluid attenuated inversion recovery (FLAIR) T2 images on axial **(A)** and coronal planes **(C)** showing high signal intensities in parasagittal subcortical parietal regions (indicated by the arrows). Follow up after 6 days showing a complete resolution of the lesions on axial **(B)** and coronal planes **(D)**.

On the 4^th^ day of hospitalization, the patient developed severe headache associated with increased blood pressure up to 150/120 mmHg (stage 2 hypertension: > 99th percentile for gender, age and height plus 5 mmHg [5]). Alterations of conscious level, visual symptoms and vomiting were not present. Furthermore, cognitive dysfunctions, visual field defects, sensory abnormalities and ataxia were absent on the neurological examination.A brain MRI showed in fluid attenuated inversion recovery imaging (T2 coronal) high signal intensities in cerebellar white matter (Figure 1A and C) and in parasagittal subcortical parietal regions (Figure 2A and C). Diffusion weighted images did not show any restriction of diffusivity.

Methylprednisolone was discontinued. Candesartan was initiated and on 6^th^ day of hospitalization, blood pressure normalized and both headache and periorbital edemas resolved. On 9^th^ day of hospitalization, a control MRI showed complete regression of all the abnormalities (Figures 1B, D and 2B, D). Arterial hypertension, MRI findings and clinical outcome were consistent with the diagnosis of PRES.

The child was discharged in good clinical condition without any further treatment.

3 weeks later, clinical examination was unremarkable and blood pressure 105/70 mmHg. Complement C3 and serum creatinine normalized and urinalysis revealed only persistent isolated microscopic hematuria (7 cells per high-power field).

## Discussion

Posterior reversible encephalopathy syndrome has often been reported in pediatric cases associated with hypertension due to renal diseases or steroid treatment [3,6].

Symptoms usually include seizures, mental status changes, visual alterations, headache and vomiting [6,7].

The literature describes posterior regions of the brain being frequently involved, but concomitant anterior lesions are often detected. Brain stem, basal ganglia, deep white matter, or splenium of the corpus callosum lesions are also described in less than one third of the patients [1,8]. The pathophysiology of this syndrome is not fully understood, but appears to be multifactorial. The underlying mechanism could be a brain capillary leak syndrome following hypertension, fluid retention or cytotoxic effects of immunosuppressive agents on the vascular endothelium. A sudden rise in blood pressure is probably the most common cause. It can induce disruption of cerebral vascular auto-regulation, mostly in the posterior cerebral vasculature, causing leakage of fluid into the brain parenchyma [6-10]. Indeed a gradual blood pressure control is recommended to avoid cerebral hypoperfusion and increased morbidity [11].

Moreover this syndrome can be irreversible or even fatal if an appropriate treatment is not started promptly [11].

## Conclusions

To the best of our knowledge, severe and isolated headache and hypertension have never been reported as unique clinical presentation of PRES. We speculate that the prompt recognition and treatment of this syndrome prevented its progression to the full blown syndrome.

### Consent

The parents signed a case report consent for publication.

## Abbreviations

MRI: Magnetic resonance imaging; PRES: Posterior reversible encephalopathy syndrome.

## Competing interests

The authors declare that they have no competing interests.

## Authors’ contribution

GPM prepared the first draft and the last version of the manuscript and approved the final manuscript as submitted. AE had responsibility for the management of the patient, wrote the first draft of the manuscript, prepared the final draft of the manuscript and approved the final manuscript as submitted. GT reviewed the literature and approved the final manuscript as submitted. EA had responsibility for the management of the patient and approved the final manuscript as submitted. CMC had primary responsibility for the management of the patient and approved the final manuscript as submitted. EAL had primary responsibility for the management of the patient and approved the final manuscript as submitted. EL wrote the first draft of the manuscript and approved the final manuscript as submitted. AMC had responsibility for the management of the patient, wrote the first draft of the manuscript and approved the final manuscript as submitted. CA wrote the first draft of the manuscript and approved the final manuscript as submitted. EFF had primary responsibility for the management of the patient, wrote the first draft of the manuscript, reviewed the literature and approved the final manuscript as submitted.

## Pre-publication history

The pre-publication history for this paper can be accessed here:

http://www.biomedcentral.com/1471-2431/14/190/prepub
